# A Role of Multi-Omics Technologies in Sheep and Goat Meats: Progress and Way Ahead

**DOI:** 10.3390/foods12224069

**Published:** 2023-11-09

**Authors:** Jin Wang, Yu Fu, Tianyu Su, Yupeng Wang, Olugbenga P. Soladoye, Yongfu Huang, Zhongquan Zhao, Yongju Zhao, Wei Wu

**Affiliations:** 1 College of Animal Science and Technology, Southwest University, Chongqing 400715, China; 2 Chongqing Key Laboratory of Herbivore Science, Southwest University, Chongqing 400715, China; 3 College of Food Science, Southwest University, Chongqing 400715, China; 4 Lacombe Research and Development Centre, Agriculture and Agri-Food Canada, Government of Canada, 6000 C&E Trail, Lacombe, AB T4L 1W1, Canada

**Keywords:** sheep, goat, meat quality, transcriptomics, proteomics, metabolomics

## Abstract

Sheep and goat meats are increasingly popular worldwide due to their superior nutritional properties and distinctive flavor profiles. In recent decades, substantial progress in meat science has facilitated in-depth examinations of ovine and caprine muscle development during the antemortem phase, as well as post-mortem changes influencing meat attributes. To elucidate the intrinsic molecular mechanisms and identify potential biomarkers associated with meat quality, the methodologies employed have evolved from traditional physicochemical parameters (such as color, tenderness, water holding capacity, flavor, and pH) to some cutting-edge omics technologies, including transcriptomics, proteomics, and metabolomics approaches. This review provides a comprehensive analysis of multi-omics techniques and their applications in unraveling sheep and goat meat quality attributes. In addition, the challenges and future perspectives associated with implementing multi-omics technologies in this area of study are discussed. Multi-omics tools can contribute to deciphering the molecular mechanism responsible for the altered the meat quality of sheep and goats across transcriptomic, proteomic, and metabolomic dimensions. The application of multi-omics technologies holds great potential in exploring and identifying biomarkers for meat quality and quality control, thereby promoting the optimization of production processes in the sheep and goat meat industry.

## 1. Introduction

Sheep and goat meats constitute a critical component of global meat consumption. The global production of goat and sheep meats has witnessed a remarkable increase in the recent years. Sheep and goat meats are of paramount global significance due to the exceptional nutritional density and distinctive flavor of sheep and goat meats, making them a pivotal component of the worldwide food landscape. The gastronomic quality of sheep and goat meats is the result of various complex factors. The color, tenderness, flavor, and water-holding capacity (WHC) of sheep and goat meats are the primary determinants of their sensory quality. In general, different breeds have different potential to produce meat [1]. Meat quality varies between different body parts within the same breed [2]. In addition, feeding management [3], transportation, slaughtering processes [4], and processing methods [5] can also influence the meat quality. The traditional sheep and goat meat quality parameters include color, pH, tenderness, WHC, and flavor. These indices can be used to detect whether the deterioration of meat quality occurs, but they cannot be employed to control and produce high-quality meat. Therefore, it is urgent to understand the molecular mechanism responsible for the altered meat quality of sheep and goats. Unfortunately, there is still a dearth of objective methods for evaluating the palatability of sheep and goat meats. Hence, it is crucial to understand the technical principle of approaches used for the analysis of sheep and goat meat quality, improve the accuracy to better reflect the changed quality, and elucidate the molecular mechanism responsible for high-quality meat [6].

High-throughput sequencing and high-resolution mass spectrometry have enabled extensive multi-omics research that can comprehensively and systematically elucidate meat quality mechanisms at the molecular level. The omics technologies commonly used to study meat quality mainly include genomics, transcriptomics, proteomics, metabolomics, etc. Genomics is the study of the total or part of the genetic or epigenetic sequence data in organisms, aiming to elucidate both the structure and function of these sequences and their consequential biological manifestations. Transcriptomics entails the systematic exploration of the transcriptome, which represents the entirety of RNA molecules that exist within a specified sample, such as a cells, tissues, or organs, at a specific moment. Proteomics is the comprehensive profiling of the holistic protein complement of a cell line, tissue, or organism. Metabolomics is the systematic identification and quantitation of all the metabolites within a specified organism or biological sample.

Due to the inherent genetic characteristics and emphasis on the role in genetic processes, genomics is normally used to calculate the heritability of goat/sheep meat quality. Intramuscular fat (IMF) is highly heritable and greatly influences meat quality, making it a key focus in genomic studies. The estimates of heritability and genetic associations reveal that IMF has high-level heritability in sheep and strongly correlates with the tenderness, WHC, and toughness of lamb meat. These findings indicate that breeding values can be utilized to select a superior meat quality by targeting the IMF content [7]. Previous studies have also supported this viewpoint [8,9,10,11,12]. Notably, the IMF content in lamb meat can exert significant positive effects on the meat color, greatly enhancing its redness and consumer desirability [13]. However, the excessive pursuit of a rapid growth rate during breeding may adversely affect the meat color, making it more susceptible to discoloration [14]. In addition, genomics has revealed a moderate heritability for cooking loss and shear strength, which are two indicators of lamb quality based on the identification of different haplotypes [15]. By summarizing the application of genomics to lambs, it is not difficult to find that the genomic approach is mainly applied in breeding to improve the meat quality, but implementation usually takes a long time. Hence, our present work mainly focuses on the research progress of post-genomic technologies, namely transcriptomics, proteomics, and metabolomics, to investigate goat/sheep meat during the post-mortem stage.

Multi-omics technologies are robust in exploring the genes’, proteins’, and metabolites’ underlying variations in meat quality. The identified biomolecular signatures can serve as biomarkers to predict quality attributes and illuminate the molecular interplay. With such technologies, recent studies have successfully explored the impacts of various physiological processes on meat quality during meat production [16,17]. However, compared to beef and pork, reviews summarizing the application of multi-omics technologies specifically for sheep and goat meat research remain scarce.

In this regard, this review focuses on the following aspects. Firstly, the technological principles of transcriptomics and their application in research on sheep and goat meats are introduced to understand the impacts of genes in different breeds and feeding management on intramuscular fat and muscle fiber development. The second section discusses the principles of proteomics technologies and related application in sheep and goat meats and explains the effects of pre- and post-slaughter factors on meat at the proteomic level. In the following section, the major principles of metabolomics technologies and their application to sheep and goat meats are reviewed. Some differentially expressed metabolites that cause changes in flavor and tenderness are summarized. In addition, this paper introduces the application of multi-omics in research on sheep and goat meats, focusing on the integrated methodological study of biological systems. Eventually, the main challenges and future perspectives of multi-omics technologies in the study of sheep and goat meat quality are discussed.

## 2. Transcriptomics

### 2.1. Overview of Transcriptomics

Transcriptomics focuses on the transcription and regulation of mRNA and ncRNA in cells, tissues, or individuals under specific conditions. ncRNA is divided into miRNA, IncRNA, and circRNA and regulates the initiation and post-transcriptional modification of mRNA transcription. It is well known that RNA is involved in muscle growth and development, muscle fiber type transformation, and intramuscular fat deposition [18,19,20]. For instance, the muscle regulatory factor family, myostatin, and insulin-like growth factor family play a regulatory role in muscle growth. Therefore, it is of great significance to explore the influencing factors of meat quality using transcriptomics via detecting the spatiotemporal specific expression of genes under different states to explain the action mechanism responsible for the altered meat quality.

### 2.2. Transcriptomics Techniques

In recent years, transcriptomic techniques have experienced three technological stages, including reverse transcription-polymerase chain reaction (RT-PCR), gene microarray, and RNA-seq. RT-PCR converts RNA to cDNA and uses cDNA as a template for amplification; the expression of the target gene after amplification is detected according to the fluorescence in the reaction tube [21]. By contrast, gene microarray technology is based on the principle of complementary base pairing. cDNA probes are immobilized on the matrix using either inkjet technology or a lithography mask [22]. An abundance of RNA was determined according to the fluorescence intensity before and after the reaction of test sample with cDNA on the matrix [23]. However, as for the abundant genes, there is a threshold for microarray technology. Regarding the genes with low-level expression, it is difficult to detect them, as the fluorescence signal may be overwritten. Moreover, unknown splicing variations cannot be detected in the presence of high-level background noise [24]. In addition, RNA-seq is a state-of-the-art technology used to solve the above shortcomings. Since its introduction in 2008, the third-generation sequencing technology has experienced progressive development. It employes the capabilities of high-throughput sequencing methods to provide insights into the transcriptome of a cell, involving RNA extraction, RNA fragmentation, cDNA generation, library amplification, and sequencing [25,26]. The first-generation low-throughput sequencing technologies are produced by Sanger. Second-generation sequencing achieves the advantages of high-throughput sequencing through chain polymerization and bridge amplification with the addition of connectors. Due to the differences between different strands in the replication process, short fragments are preferred. The third-generation sequencing technology directly connects sequenced RNA molecules into loops for sequencing to achieve ultra-long read lengths. However, the accuracy of third-generation sequencing needs to be improved; thus, second-generation sequencing is currently the most widely used due to its high-throughput advantages.

### 2.3. Application of Transcriptomics in Sheep and Goat Meat Research

At present, a series of studies have employed transcriptomics to analyze meat quality traits and explore the genes with the potential to produce an excellent meat quality. A number of recently advanced transcriptomic technologies applied for the analysis of the *Longissimus dorsi* muscle and *Longissimus thoracis* muscle from goats and sheep are summarized in Table 1.

#### 2.3.1. Differences in IMF Deposition across Diverse Breeds

The proportion and composition of IMF are important indicators for evaluating the meat quality, which directly can affect the tenderness, meat color, juiciness, and flavor [5]. Kumar et al. (2021) [1] used RNA-seq technology to analyze the transcriptomes of the *Longissimus pectoralis* muscles of Barbari goats renowned for a high-quality meat and Changthangi goats renowned for wool. In total, 3120 differentially expressed genes were identified, of which 1492 were up-regulated and 1628 were down-regulated in the Barbari goats. The up-regulated genes in the Barbari goats were mainly involved in the synthesis of fatty acid, triglyceride catabolism, gluconeogenesis, ion transport, transporter activity, etc. For instance, in terms of the differentially expressed genes in fatty acid synthesis, the *FASN* gene can catalyze the synthesis of long-chain fatty acids, while the *FABP3* gene is mainly involved in cell metabolism and transport of long-chain fatty acids [27]. The *SCD* gene encodes stearoyl-CoA desaturase that converts trans fatty acids (C18:1) to conjugated linoleic acid (CLA) [28]. In the triglyceride metabolic pathway, the *GPAT* gene can catalyze glycerol phosphate and lipyl-coenzyme A (CoA) to produce lysophosphatidic acid (LPA), the *AGPAT* gene contributes to the production of diacylglycerol (DAG), and the *DGAT* gene produces triacylglycerol (TAG) in the final step, which is deposited between the muscle fibers and muscle bundles to form intramuscular fat [29]. In contrast, in Changthangi goats, the highly expressed genes were mainly involved in muscle contraction [1], which indicated the difference in meat quality between the meat goats and hair goats. Cheng et al. (2020) [30] also used RNA-seq technique to identify 874 differentially expressed genes in the *Longissimus dorsi* muscles of Dorper × Small-tailed Han sheep and Small-tailed Han sheep, among which 32 genes were related to meat quality. These genes mainly involve lipid metabolism, energy decoupling in skeletal muscles, and the homeostasis regulation of bones or muscles. These processes can promote IMF deposition, so as to improve the meat quality via cross breeding. Peng et al. (2022) [31] used transcriptomes to analyze the *Longissimus dorsi* of Dorper sheep and Small-tailed Han sheep, and the results showed that *FABP3* and *LPL* (lipoprotein lipase) in Dorper sheep meat were significantly up-regulated. The *FABP3* gene has the same function as described earlier, promoting esterification reactions and synthesizing triglycerides. *LPL* promotes the decomposition of Chylomicron into triglycerides and lipoproteins/phospholipids, which contributes to IMF deposition in Dorper sheep.

#### 2.3.2. Effects of Different Feeding Managements on IMF Deposition

Not only breed can affect the IMF content, but also, the difference in feeding management affects fat deposition. For example, An et al. (2021) [32] collected *Longissimus dorsi* from sheep fed different diets for transcriptomics analysis. A total of 443 differentially expressed genes were identified, including 169 up-regulated genes and 274 down-regulated genes. The biological processes affected by differentially expressed genes involved IMF and fatty acid deposition and triglyceride transport. For example, the up-regulated *ACSL1* gene can retard the β-oxidation of fat via the PPARγ signaling pathway, thereby increasing the level of triglyceride [33]. The *FABP3* gene can specifically bind to free fatty acids and transport intracellular fatty acids to certain sites, where triglycerides and phosphoesters are synthesized or degraded, facilitating esterification reactions and triglyceride resynthesis, thereby increasing the content of intramuscular fat [34]. Zhang et al. (2022) [35] used the transcriptome to analyze the *Longissimus thoracis* of Sunit sheep fed probiotics and control groups. The results showed that *MYOD1* inhibited the adipogenic differentiation of skeletal myosatellite cells and promoted the differentiation of muscle cells and myotube formation. Zhao et al. (2022) [36] found that the levels of 4-methyloctanoic acid and 4-methylnonanoic acid in lambs fed scallions were significantly lower than those in the control group, resulting in a significant reduction in mutton odor. Transcriptomics analysis showed that the pathways involved were glutathione metabolism, extracellular matrix receptor interaction and steroid hormone biosynthesis. Recently, Su et al. (2019) [37] have collected the *Longissimus dorsi* of Sunit sheep that perform different levels of endurance exercise for transcriptomics analysis, and the differentially expressed genes were identified to be related to glycolysis, which demonstrates that endurance exercise increased the rapid glycolysis fiber content and glycolysis potential of the meat, thereby affecting the color of the meat. Wen et al. (2022) [38] analyzed the transcriptome in the *Longissimus thoracis* muscles of Tibetan sheep at different ages; the sheep meat in the 1.5-year-old group was the most tender, with a higher content of type I muscle fibers, thus containing more lipids. Meanwhile, acetyl CoA carboxylase (ACC1) was highly expressed in this group, and its catalytic product propionyl CoA promoted mitochondrial biogenesis, meeting the requirements for type I muscle fiber development.

**Table 1:** Application of transcriptomics in studies of sheep and goat meats.

Breed (Species)	Technology	Muscle/ Tissue	Treatments	Factors	Meat Traits	Ref.
Barbari and Changthangi goats (*Capra hircus*)	RNA-Seq (Illumina)	*Longissimus thoracis*	Four Barbari goats and four Changthangi goats with the same growing environment and feeding management were selected.	Breed	IMF content	[1]
Dorper × Small-tailed Han sheep (*Ovis aries*)	RNA-Seq (Illumina)	*Longissimus dorsi*	Fifty Dorper × Small-tailed Han and fifty Small-tailed Han sheep were fed 3 times a day (6 a.m., 12 p.m., 18 p.m.) in the same environment for 6 months.	Breed	Color and tenderness	[30]
Dorper and Small-tailed Han sheep (*Ovis aries*)	RNA-Seq (Illumina)	*Longissimus dorsi*	Dorper and Small-tailed Han sheep with similar age and weight were selected and fed for 6 months under the same feeding management.	Breed	Tenderness	[31]
Dorper sheep (*Ovis aries*)	RNA-Seq (Illumina)	*Longissimus dorsi*	Twelve Dorper × Hu crossbred weaning male sheep were fed with different proportions of fermented diets. The mixed proportions of Broussonetia papyrifera L treated with fermented feed were 0%, 6%, 18%, and 100% of the total diet.	Feeding manage-ment	Tenderness	[32]
Sunit sheep (*Capra hircus*)	RNA-Seq (Illumina)	*Longissimus thoracis*	Twelve 3-month-old Sunit lambs were divided into two groups. The experimental group was supplemented with 1% probiotic feed and fed for 90 days.	Feeding manage-ment	/	[35]
Small-tailed Han sheep (*Ovis aries*)	RNA-Seq (Illumina)	*Left hepatic lobe*	Thirty 3-month-old Small-tailed Han sheep were randomly divided into two groups. The experimental group was supplemented with allium mongolicum extract. After 75 days of feeding, 12 sheep in each group were randomly selected for slaughter.	Feeding manage-ment	Flavor	[36]
Sunit sheep (*Capra hircus*)	RT-PCR	*Longissimus dorsi*	Twenty-four Sunit sheep with the same genetic background were randomly divided into two groups, namely free grazing group and house feeding group.	Feeding manage-ment	Color	[37]
Tibetan sheep (*Ovis aries*)	RNA-Seq (Illumina)	*Longissimus thoracis*	The meat samples from Tibetan sheep aged 4 months, 1.5, 3.5, and 6 years were collected from the same feeding environment.	Growth stages	Tenderness	[38]

**Figure 1:**
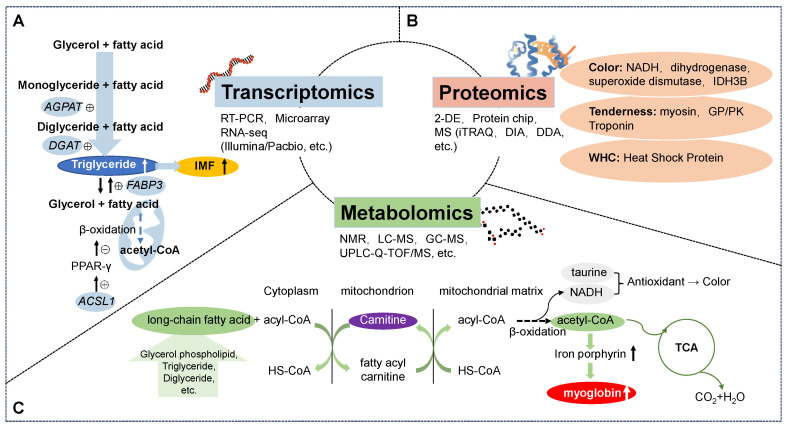
(**A**) Transcriptomics platforms and pathways involved in IMF deposition in sheep/goat meat research. (**B**) Proteomics platforms and biomarkers related to sheep/goat meat quality. (**C**) Metabolomics platforms, metabolic pathways, and biomarkers related to sheep/goat meat quality.

Taken together, previous transcriptomic studies on sheep/goat meat quality have mainly focused on IMF deposition, and the specific pathways involved are outlined in Figure 1A. The application of transcriptomics in the study of sheep and goat meats is robust in identifying the differentially expressed genes, their functions, and the related signaling pathways. This contributed to elucidating the molecular mechanisms underlying phenotypic changes. The differentially expressed genes related to meat quality traits, such as tenderness and flavor, exhibit a moderate genetic capacity [39], which is of great significance for breeding sheep and goats with an excellent meat quality.

## 3. Proteomics

### 3.1. Overview of Proteomics

The proteome refers to the sum of all the proteins expressed in tissues or living organisms at a particular time and space. Proteomics is a visual description of the expression and function of the proteome, using a variety of techniques to identify and determine the types and abundance of proteins, protein–protein interaction, the localization of proteins in cells, and post-translational modification [40]. Among them, protein translational modifications (PTMs) include acetylation, phosphorylation, ubiquitination, methylation, and glycosylation modification, which are involved in protein stability, regulating protein activity in signal transduction, protein metabolism, gene expression, and protein folding. Moreover, protein modification can rapidly regulate protein activity and function under endogenous and exogenous stimulation [41]. Proteins are important components of muscles, so various proteomic changes can take place during the conversion process from muscle to meat. Therefore, proteomics technology can be employed to ascertain biomarkers of meat quality.

### 3.2. Proteomics Techniques

With the development of biotechnology, proteomics technologies have gone through three stages, namely two-dimensional gel electrophoresis and protein chip and mass spectrometry-based proteomics.

Currently, mass spectrometry-based proteomics has been widely applied to identify proteins, post-translational modifications, subunit interactions, and screen for the differentially expressed proteins related to the changes in food quality. Accurate protein quantification is necessary to better elucidate the molecular mechanisms of biochemical processes. Mass spectrometry combined with sample preparation methods provides this possibility. Various mass spectrometry-based techniques for protein quantification can be used, such as labeling-based methods, different data acquisition modes, types of mass analyzers, and bioinformatics approaches. Some examples of these methods include labeled and label-free quantification and data-dependent acquisition (DDA) and data-independent acquisition (DIA) modes (especially sequential window acquisition of all theoretical fragment ions, SWATH), as well as multiple reaction monitoring (MRM). And the current development of ‘4D’ proteomics by adding ion mobility to ‘3D’ proteomics can improve the selectivity of parent ions for more accurate separation.

### 3.3. Application of Proteomics in Sheep and Goat Meat Research

Proteomics technologies have been used to search, screen, and identify the proteins related to meat quality, providing a direction for the accurate evaluation and rapid monitoring of meat quality. The recent progress in applying proteomics to study the tenderness, color, and WHC of goat and sheep meats is summarized in Table 2. The influence of various factors, including the growth environment, slaughter method, storage, processing, and post-mortem protein modification, on goat and sheep meat quality has become the main research focus.

#### 3.3.1. Changes in Meat Quality during Storage

Since freezing is the most common long-term storage method for meat, it is particularly important to explore the changes in goat and sheep meat quality during frozen storage. Gu et al. (2020) [42] utilized the isobaric tag for relative and absolute quantitation (iTRAQ) technology for the proteomics analysis of goats’ *Longissimus thoracis* muscles during repetitive freeze–thaw cycles. They identified 29, 127, and 133 differentially expressed proteins (DEPs) at three storage time points, respectively, which are mainly muscle fiber component proteins, while repeated freeze–thaw cycles led to muscle fiber breakage, a decreased WHC, and tenderness. However, the up-regulated expression of NADH dehydrogenase and IDH3B after the first freeze–thaw cycle promoted myoglobin reduction from metmyoglobin, improving the meat color stability. These results provided a basis to determine the freezing times for cold chain transportation. Jia et al. (2021) [43] collected the *Longissimus lumborum* of Hengshan goats and froze the muscles for 0, 30, and 60 days, respectively. Compared to day 0, a total of 166 DEPs were identified in the meat on day 30, with 156 down-regulated and 10 up-regulated ones. On day 60, 84 of 98 DEPs were down-regulated, and 14 were up-regulated. Enrichment analysis showed adenosine triphosphate isomerase and peroxide dismutase could potentially serve as biomarkers for meat color. Troponin and myosin were associated with tenderness. Heat shock proteins retained water in the meat, while the glycolysis and ubiquitin–proteasome of DEPs led to long-term degradation during frozen storage.

#### 3.3.2. Effects of Slaughtering Methods on Meat Quality

Slaughter practices can affect carcass metabolism after slaughter, resulting in differences in the meat quality. Kiran et al. (2021) [44] analyzed fifteen sheep slaughtered by sending an electric shock through their heads or in another way. Proteomics analysis was performed on the *Longissimus thoracis et lumborum* muscle after rigor mortis. There were 35 DEPs identified (26 up-regulated and 9 down-regulated). The expression levels of antioxidant, binding, chaperone, and heat shock proteins increased in the non-shocked sheep meat, resisting cell apoptosis. In contrast, the electric shock group had increased metabolic protein expression, facilitating lactic acid consumption and ultimately increasing the pH to a certain extent. Therefore, the DEPs after these two treatments may be used as animal welfare indicators. Similarly, Hosseini et al. (2019) [45] found that ethical slaughtering exerted a positive impact on maintaining heme integrity and high-level antioxidant activity, suggesting that the slaughtering practices significantly impact the meat color.

#### 3.3.3. Effects of Processing Methods on Meat Quality

Thermal processing can induce protein oxidation, which affects the texture of the meat. Researchers collected the *Longissimus* of Hengshan goats and performed the DIA-based comparative proteomics analysis of goat meat after boiling, roasting, steaming, and no treatment. Compared to the control, 101, 98, and 109 proteins were differentially expressed in each treatment. These DEPs included structural and metabolic proteins. Myosin was more likely to undergo oxidation modification during grilling, leading to tougher meat. Boiling reduced the amount of troponin T, but increased the content of glycolytic enzymes, improving the tenderness of the meat. Combined with sensory evaluation, it is suggested that boiling is an effective thermal treatment method to ensure the tenderness of goat meat [46].

#### 3.3.4. Effects of Post-Mortem Protein Modification on Meat Quality

The varying degree of post-mortem glycolysis affects meat tenderness [47]. Li et al. (2017) [48] collected the *Longissimus thoracis et lumborum* muscles of 6-month-old non-castrated sheep. Quantitative phosphoproteomics analysis identified 5, 8, and 9 phosphoproteins in ovine muscles with different degrees of tenderness over time (at 0.5, 4, and 24 h post mortem). These phosphoproteins were mainly involved in the glycolysis pathway. For example, pyruvate kinase (PK) is a rate-limiting enzyme in the glycolytic pathway, catalyzing phosphoenolpyruvate to pyruvate and ATP. Phosphorylated PK is highly active, so the high level of phosphorylation promotes glycolysis, resulting in tougher meat [49]. Chen et al. (2018) [50] also indicated that protein phosphorylation regulated the glycolysis rate and meat quality. Moreover, glycolytic enzyme activity, calpain proteolysis, Z-disk protein degradation, myoglobin stability, and actomyosin dissociation were involved. All these proteins were regulated via phosphorylation [51].

**Table 2:** Application of proteomics in studies of sheep and goat meats.

Breed (Species)	Technology	Muscle	Treatments	Factors	Meat Traits	Ref.
*Capra hircus*	iTRAQ	*Longissimus thoracis*	The meat samples were collected after 0, 1, and 2 freeze–thaw cycles, respectively.	Freeze–thaw cycles	Color and tenderness	[42]
Hengshan goats (*Capra hircus*)	DIA	*Longissimus lumborum*	After frozen for 0, 30, and 60 days, the meat samples were collected for proteomics analysis.	Freezing times	Color and tenderness	[43]
Nellore sheep (*Ovis aries*)	2-DE/MS	*Longissimus thoracis et lumborum*	Fifteen Nellore sheep were slaughtered without prior electrical stunning, and the other sheep were electrically stunned.	Slaughtering method	Ultimate pH and tenderness	[44]
Hengshan goats (*Capra hircus*)	DIA	*Longissimus*	The meat samples were collected after boiling, roasting, and steaming processing.	Thermal processing	Tenderness	[46]
Fat-tailed sheep × Small-tailed Han sheep (*Ovis aries*)	2-DE/MS	*Longissimus thoracis et lumborum*	Forty 6-month-old, uncastrated sheep were collected at 0.5, 4, 12, and 24 h post mortem.	Storage time	Tenderness	[48]

To sum up, as illustrated in Figure 1B, proteomics has been widely used to study sheep/goat meat quality and identify relevant biomarkers. Conducting proteomic research on meat quality can provide insights to guide animal breeding and production for a superior meat quality [52]. 

## 4. Metabolomics

### 4.1. Overview of Metabolomics

A metabolome is a complete set of metabolites with a molecular weight under 1000 Da in a biological sample. Metabolomics normally employs high-throughput technology to qualitatively and quantitatively analyze the metabolites that respond to environmental changes, revealing metabolic mechanisms [53]. During meat production, metabolites change with different breeds, feeding, and storage conditions. Correlating metabolites to biological phenotypes can help improve the meat quality by analyzing the metabolites and quality changes.

### 4.2. Metabolomics Techniques

Metabolomic techniques mainly include nuclear magnetic resonance (NMR), gas chromatography–mass spectrometry (GC-MS), and liquid chromatography–mass spectrometry (LC-MS), with different characteristics. NMR can be used to understand the state of nutrients in meat quality by measuring the molecular relaxation time, followed by distinguishing the meat quality. GC-MS is used to measure volatile compounds, such as aldehydes, ketones, and alcohols. Moreover, amino acids, fatty acids, and other non-volatile compounds can be detected via LC-MS. 

### 4.3. Application of Metabolomics in Sheep and Goat Meat Research

With advances in metabolomics, a robust workflow has been developed for identification. Samples undergo pre-treatment; then, NMR, GC-MS, or LC-MS is selected based on the required detection. Finally, data analysis and visualization are performed. Currently, metabolomics has preliminarily studied how preslaughter factors (e.g., breed, feeding) and post-mortem factors (e.g., storage) affect goat and sheep meat quality, as summarized in Table 3.

#### 4.3.1. Strategies for Improving Meat Quality Based on Metabolomics 

Different breeds have varying potentials for the production of high-quality meat. Other researchers carried out studies on the differentially expressed metabolites among breeds. The LC-MS-based metabolomics analysis of three goat breeds (Lubei white, Jining grey, and Boer goats) identified 977 metabolites in the *Latissimus dorsi* muscle. While the Jining grey goat meat contained abundant pleasant flavor metabolites and fewer bitter alkaloids, conferring superior sensory properties, the Boer goat meat was characterized by arachidonic acid imparting a pungent and sweet taste. The higher levels of polyunsaturated fatty acids and less-saturated long-chain carboxylates in Lubei white goat muscles resulted in a more bitter taste [54]. Kosowska et al. (2017) [55] also indicated that polyunsaturated and monounsaturated fatty acids may be oxidized during cooking, leading to flavor defects. 

The meat quality can be improved by adjusting the diet composition. Grazing is an important approach to alter diet patterns. Wang et al. (2021) [3] randomly assigned 24 Tan sheep (aged 120 days) into three groups, namely indoor feeding (F), grazing with indoor feeding (GF), and pure grazing (G). The GC-MS metabolomics of the *Psoas* muscle identified 33, 70, and 61 differential metabolites among the respective groups, mainly involving amino acid, lipid, and nucleotide metabolism. Group G showed increased acetylcarnitine, L-carnitine, and N-acetylaspartic acid contents, improving the meat color by regulating myoglobin synthesis. Decreased carnosine and creatinine contents reduced the bitter and sour taste intensity. The GF group had more sweet and umami amino acids, acting as precursors affecting the meat flavor. 

Currently, consumers prefer chilled meat due to their changing habits. Thus, the metabolite changes during meat storage warrant further study. You et al. (2018) [56] stored Tan sheep hind leg meat at 0 °C for 8 days. The metabolites correlating with meat freshness were identified as biomarkers. Among the 27 differentially abundant metabolites, glycogen and protein degradation occurred on day 4, while the alanine, aspartate, and glutamate synthesis levels increased. On day 8, pathways, including the TCA cycle, amino sugar biosynthesis, and nucleotide metabolism, activated, producing 1,5-dehydrated glucitol. This significantly reduced the pH, increased the number of endogenous proteases, and accelerated spoilage. These data help characterize and predict meat freshness. Overall, metabolomics techniques have a significant effect in explaining the effects of breed, feeding, and storage time on meat metabolites, which is of great significance in improving meat quality.

#### 4.3.2. Applications of Lipidomics and Flavor Metabolomics in Improving Meat Quality

Lipidomics plays an important role in revealing the lipid categories, abundance, spatial distribution, and cellular lipid metabolism. Flavor metabolomics focuses on volatile compounds and amino acids [57]. As branches of metabolomics, lipidomics and flavoromics apply similar platforms. It is worth noting that lipids and volatiles require prior extraction. Given their high lipid content, sheep and goat meats are prone to oxidation during production and storage. Also, gaminess may limit sales. Thus, lipidomics and flavor metabolomics are crucial for sheep and goat meats. 

The nutritional ratio of feed significantly can affect high-quality lamb meat production. The flavor of lamb relates to lipids, and high-energy/high-fat diets promote lamb lipid synthesis. Wang et al. (2021) [58] analyzed the Biceps femoris from pasture- and concentrate-fed sheep/goats using metabolomics and lipidomics. They found that pasture feeding produced more inosine monophosphate (IMP), a better flavor, more taurine, and increased the color stability, while concentrate-fed meat has higher levels of L-carnitine and acetyl-carnitine, with more lipid deposition. Li et al. (2022) [59] applied lipidomics to the *Longissimus lumborum* of Hu sheep with high and low levels of IMF, identifying 79 differential lipids. Meat with low-level IMF is associated with lipid derivatives, such as 1-octen-3-ol, 2-pentyl furan, nonanal, heptanal, and butanal. Meat with high-level IMF positively correlate with dimethyl trisulfide. Fatty acid analysis revealed that linoleic acid may react with Maillard products during processing, producing meaty flavors. In addition, both nutrition and management influence IMF deposition. Li et al. (2020) [60] conducted lipidomics on the *Psoas major* of castrated male Hu sheep. Castration decreased the level of sex hormones, while increasing the IMF content. Accordingly, castration reduced the quantity of ketones, but increased the contents of alcohols, aldehydes, and esters versus those of the controls, thereby improving the lambs’ flavor.

High-fat sheep and goat meats have a unique flavor profile, but antioxidant preservation during refrigerated storage remains challenging. Further research is needed to thoroughly investigate storage methods and characterize the lipid changes under various storage conditions. Normally, refrigeration is generally the safest and most accessible storage approach for both meat producers and consumers. Xu et al. (2023) [61] collected the left *Longissimus dorsi* muscles from Tan sheep and stored them at 4 °C. Lipidomics analysis was performed on storage days 0, 1, 3, 5, and 7 to identify 20 volatile compounds and 2616 lipids. On the third day of storage, the levels of 1-octen-3-ol, 6-methyl-2-heptanone, and 3-heptanone increased, endowing the meat with a floral, fruity, or creamy flavor, respectively. Changes in acylcarnitines and triglycerides indicated that fatty acid β-oxidation contributed to flavor differences over time. Zhang et al. (2023) [62] identified 95 differential lipids in Mongolian sheep meat stored at 4 °C using lipidomics. Glycerophospholipids and fatty acyls were the major lipids altered due to oxidation, increasing the quantity of polyunsaturated fatty acids (PUFA). PUFA accumulation promoted mitochondrial uncoupling, increasing the β-oxidized acylcarnitine content, generating heat, and declining the meat quality. Jia et al. (2021) [63] also performed lipidomics analysis on Ningxia Tan sheep meat during frozen storage (−20 °C). In the first 12 days of storage, the ratio of long-chain acylcarnitines to L-carnitine increased, boosting fatty acid oxidation. Over the next 12 days, fatty acid oxidation decreased, while phospholipids converted to sphingolipids, increasing the rate of self-oxidation. The content of L-(+)-cysteine increased throughout the storage period, leading to an increase in the bitterness of meat. 

Preservatives are also an important antioxidant approach during sheep and goat meat storage. Jia et al. (2021) [64] treated the *Longissimus thoracis et lumborum* of Tan sheep with nisin and potassium sorbate preservatives, and then stored them at 4 °C and identified 106 significantly changed lipids. Potassium sorbate preservatives led to a decrease in the meat pH, inhibited phospholipase C production, and reduced the amount of diacylglycerol. The nisin group’s lipid profile did not differ significantly from that of the control, making it an ideal preservative. Irradiation can inhibit surface bacteria, exhibit antioxidant properties, and improve the retention of nutritional value in meat. Jia et al. (2021) [65] conducted lipidomics analysis on the *Longissimus dorsi* of Hengshan goats after a gamma-irradiated treatment, identifying 174 lipids with significant differences (*p* < 0.05). The irradiated group had higher docosahexaenoic acid (DHA) levels in the phospholipids compared to those of the control, suggesting the enhanced preservation of nutritional components.

Different cooking methods can affect the lipid composition in sheep and goat meats during processing. Jia et al. (2021) [66] used lipidomics to identify 90 differential lipids in Ningxia Tan sheep *Longissimus dorsi* muscles after steaming, boiling, and roasting treatments. Boiling promoted phosphatidylcholine degradation into sphingomyelin. Steaming resulted in less loss of phosphatidylcholine and lysophosphatidylcholine, which benefits brain development and memory. Liu et al. (2022) [67] studied baking effects (15 min) on the lipid profile of sheep back strap muscles by performing lipidomics analysis. A total of 488 differential lipids were identified, among which 61 were involved in forming aromatic compounds during roasting. Triglyceride (TG) (16:0_18:1_18:1) and TG (18:0_18:0_18:1) had the highest contents, associated with the aroma of sheep meat. The contents of phosphatidylcholine (PC) (30:6) and PC (28:3) decreased sharply at 0–5 min, increased significantly at 5–7.5 min, and then decreased again at 7.5–15 min. They can serve as molecular biomarkers for roasted sheep meat.

**Table 3:** Application of metabolomics in studies of sheep and goat meats.

Breed (Species)	Technology	Muscle	Treatments	Factors	Meat Traits	Ref.
Lubei White, Jining Gray, and Boer goats (*Capra hircus*)	LC-MS	*Latissimus dorsi*	Minimally invasive muscle biopsy under local anesthesia in 6-month-old goats of three different breeds was performed.	Breed	Flavor	[54]
Tan sheep (*Ovis aries*)	LC-MS	*Lumborum*	Twenty-four Tan sheep aged 120 days were randomly divided into three groups: (1) indoor feeding (F); (2) artificial pasture grazing with indoor feeding (GF); (3) pure artificial pasture grazing (G).	Feeding management	Flavor	[3]
Tan sheep (*Ovis aries*)	GC-MS	*Hind legs*	The surfaces of refrigerated sheep hind legs were scraped on day 0, 4, and 8 in order to collect surface microbes, blood, and exudate.	Chilled storage	Freshness level	[56]
Ujimqin sheep, Sunit sheep, Small-tailed Han sheep, Boer goats, and cashmere goats (*Ovis aries/Capra hircus*)	UPLC-Q-TOF/MS	*Biceps femoris*	A total of 138 sheep/goats meat samples of two types (pasture-fed and concentrate-fed) were collected.	Feeding management	Color and flavor	[58]
Hu sheep (*Ovis aries*)	UHPLC-Q-Orbitrap MS and SPME-GC-MS	*Longissimus lumborum*	The meat samples were collected from male Hu sheep, including 12 samples with high IMF content, and 12 samples with low IMF content.	Individuality	Flavor and IMF	[59]
Hu sheep (*Ovis aries*)	UPLC Q-Exactive Orbitrap MS	*Psoas major*	Twenty-four Hu sheep with the same genetic background were divided into a castration group and a control group. The sheep were raised under the same environmental conditions for 27 weeks.	Feeding management	Flavor	[60]
Tan sheep (*Ovis aries*)	HS-SPME-GC-MS/GC-MS	*Longissimus dorsi*	The meat samples from 10 Tan sheep were packaged in plastic film and stored at 4 ± 1 °C for 0, 1, 3, 5, and 7 d.	Storage time	Flavor	[61]
Mongolian sheep (*Ovis aries*)	UPLC-ESI-MS/MS	*Longissimus thoracis*	The meat samples were collected from six Mongolian ewes and stored at 4 °C for 0, 24, 48, 72, and 96 h.	Storage time	Flavor	[62]
Tan sheep (*Ovis aries*)	UPLC-ESI-MS	*Longissimus dorsi*	The meat samples from ten 4-year-old male Tan sheep were collected, blended, and stored in PE plastic bags for up to 24 days at −20 °C.	Storing time	Flavor and eating quality	[63]
Tan sheep/Hengshan goats (*Ovis aries/Capra hircus*)	UHPLC-Q-Orbitrap MS/MS	*Longissimus thoracis et lumborum/Longissimus dorsi*	The meat samples were collected from 10 four-year-old Tan sheep and treated with different concentrations of nisin and potassium sorbate preservatives.	Preservatives	Lipid composition	[64]
Tan sheep (*Ovis aries*)	UHPLC-Q-Orbitrap HRMS	*Longissimus dorsi*	The meat samples were cooked by different methods for set times and temperatures.	Thermal processing	Lipid composition	[66]
Small-tailed sheep × Mongolian sheep (*Ovis aries*)	UPLC-ESI-MS/MS and Orbitrap Exploris GC	*back strap*	The meat samples were roasted for 0, 2.5, 5, 7.5, 10, and 15 min using the traditional charcoal method.	Thermal processing	Lipid composition	[67]

Based on the preceding discussions, Figure 1C presents an overview of the metabolic markers and pathways related to sheep and goat meat quality. Lipidomics and flavor metabolomics investigations have demonstrated the feasibility of identifying biomarkers for lamb quality. Different biomarker lipids have highlighted the key processes impacting meat quality and flavor, including unsaturated fatty acid biosynthesis, lipid oxidation, and lipid denaturation. These biomarkers can provide references to characterize meat lipids, improving our understanding of sheep and goat meat quality. However, further research and validation are needed to determine the specific functions of the lipid-related differences in meat quality.

## 5. Multi-Omics

### 5.1. Overview of Multi-Omics

**Figure 2:**
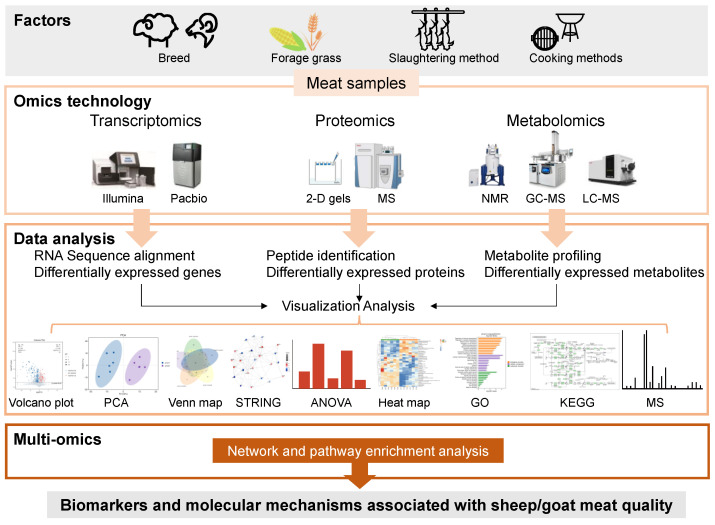
Omics strategies for sheep/goat meat quality research.

Omics utilizes high-throughput approaches to generate big data. In the post-genomic era, it consists of transcriptomics, proteomics, and metabolomics used to profile RNAs, proteins, and metabolites, respectively. Multi-omics is based on bioinformatics analysis to screen out differential expressions in big data and predict potential biomarkers. The data quality is examined before cluster analysis is used to correlate the omics data with biological phenotypes, predict functions, and map the related pathways. Interaction networks are also constructed to predict the relationships between RNAs, proteins, and metabolites [68]. Gene expression is influenced by various factors that determine the meat quality potential. However, the phenotype undergoes multiple processes, including transcription, translation, post-translational modification, and metabolism. An integrated omics strategy combining transcriptomics, proteomics, and metabolomics can help explore the expression changes during these processes, unraveling the molecular mechanisms responsible for meat quality variations. The workflow of multi-omics analysis for sheep and goat meat quality research is outlined in Figure 2.

### 5.2. Integrated Transcriptomics and Proteomics

Translation from mRNA to a protein is intricately regulated by ncRNA, cis-acting elements, trans-acting factors at the transcriptional, post-transcriptional, translational, and post-translational levels. Therefore, transcriptomic and proteomic data may not fully correlate [69,70]. However, the comprehensive characterization of key gene expression patterns remains essential in current research. Typical combined transcriptomics and proteomics analyses involve the following steps. Firstly, the mRNAs and proteins with changed expressions are correlated according to their fold change. Next, the pathway analysis of genes with consistent, opposite, or unrelated expression trends is performed to obtain integrated mRNA–protein expression patterns. Finally, the pathway and network analyses of differentially expressed genes and proteins draw on subcellular localization, biological processes, and molecular functions [71].

In a study of sheep meat quality, Zhao et al. (2022) [72] performed integrated transcriptomics and proteomics analyses on the *Longissimus thoracis* muscles of Hu and Dorper sheep. The differentially expressed genes and proteins related to lipid deposition and amino acid synthesis were identified through the joint analysis, elucidating the mechanism underlying the tender flavor and popularity of Hu sheep meat. However, such integrated analyses combining transcriptomics and proteomics are still rare in sheep and goat meat quality research and are primarily focused on IMF deposition in beef and pork [73,74]. 

Overall, integrated transcriptomics and proteomics are crucial in meat quality research, as they offer a comprehensive understanding of the molecular mechanisms governing meat quality. Transcriptomics reveals how genes are expressed, while proteomics identifies the actual proteins present in meat. By analyzing both levels simultaneously, researchers can pinpoint specific genes, proteins, and regulatory pathways that impact meat quality traits. This knowledge not only aids in understanding meat quality at a molecular level, but also guides breeding, nutritional strategies, and quality control practices in meat production, ultimately leading to better and more consistent meat products for consumers.

### 5.3. Integrated Proteomics and Metabolomics 

Unlike the central dogma between proteins and transcripts, there is no such direct relationship between proteins and metabolites. Therefore, the core of integrating proteomics and metabolomics data is pathway analysis to identify the potential regulatory associations between differentially expressed proteins (DEPs) and metabolites [75].

Callipyge sheep are important due to their increased meat yield. Ma et al. [76] collected the *Longissimus thoracis* muscles from callipyge (+/C) and non-callipyge (+/+, C/+ and C/C) lambs for proteomics and metabolomics analyses. They found that the DEPs were mainly involved in energy metabolism, apoptosis, and antioxidation. Differential metabolites also confirmed the shift from oxidation to glycolysis. These results verified that callipyge genes affected the meat quality by regulating muscle fiber type transition. Taken together, this study successfully integrated proteomics and metabolomics to comprehensively elucidate the molecular mechanisms of the callipyge genes causing variations in meat quality.

### 5.4. Integrated Transcriptomics and Metabolomics 

The metabolome can reveal the metabolic state of animals affected by genetic or exogenous factors, serving as the basis for the complex phenotypic traits of animals. The transcriptome connects to the metabolome by aggregating differentially expressed genes and a lot of regulatory pathway information.

In studying meat quality, fat deposition is an important way to improve meat tenderness, as regulated by multiple genes and signaling pathways. Wu et al. (2020) [17] explored the effects of high-fiber and low-fiber diets on lamb quality using transcriptomic and metabolomic approaches. The high-fiber diet was found to inactivate the genes in steroid signaling pathways and induce gluconeogenesis, which resulted in reduced muscle fat. The metabolomic changes also suggested that the high-fiber diet decreased fat deposition through impacts on glucose metabolism. Overall, transcriptomics and metabolomics analyses indicated that the high-fiber diet reduced the meat tenderness by decreasing fat deposition, despite promoting muscle fiber growth. Recently, transcriptomics and metabolomics have been increasingly used to study the effects of a variety of nutritional factors on sheep meat quality [57,77,78,79,80]. Overall, the integromic data obtained at different molecular levels of omics for mutual verification compensated for the flux and bias of single omics analysis. Due to the amplification effect of the genes, proteins, and metabolites, data analysis from downstream omics is more likely to explain the magnitude changes in upstream omics.

## 6. Conclusive Remarks and Future Perspective

### 6.1. Conclusive Remarks

Transcriptomics is capable of screening the differentially expressed genes for molecular breeding to obtain high-quality sheep and goat meats. Proteomics can comprehensively understand the molecular mechanism responsible for meat quality, which contributes to accurately controlling the quality of sheep and goat meats. Metabolomics is a robust high-throughput tool for the analysis of small metabolites involved in real-time changes in meat quality, which is affected by various factors. Multi-omics can better reflect the difference in meat quality in a more holistic fashion through mutual verification between multi-omics results.

In addition, integrated omics has tremendous potential in the field of sheep and goat meat research, with emerging trends and potential future directions in multi-omics investigations of meat quality, encompassing several key areas. These include the adoption of single-cell omics techniques for enhanced resolution, the exploration of epigenomics to investigate the impact of environmental factors on gene regulation, and the analysis of the microbiome to discern its influence on meat quality attributes. Additionally, the utilization of advanced artificial intelligence (AI) and machine learning methodologies for the interpretation of the vast multi-omics datasets is anticipated to become more prominent. Furthermore, research will increasingly focus on aligning meat quality with consumers’ preferences, enhancing sustainability in meat production practices, employing functional genomics, and fostering interdisciplinary collaborations to bridge the gap between the genetic potential and the sensory attributes of the final meat products. These developments are poised to facilitate more personalized and sustainable approaches to meat production, while enhancing consumers’ satisfaction and addressing societal and environmental concerns.

### 6.2. Future Perspectives in Application of Omics Technology

By summarizing the recent studies on multi-omics technology in meat quality, several challenges need to be addressed:

i. In applying omics to sheep and goat meat quality research, stable isotope tracing technology can be used to reflect the changes in intracellular substances. This would be more conducive to revealing the relevant mechanisms regulating meat quality. However, there is currently a lack of studies employing this method.
ii. As a robust tool for high-throughput screening, multi-omics research lacks reasonable verification when used for data annotation and enrichment.
iii. When multi-omics strategies are used to search for biomarkers, it is often difficult to find biomarkers for a single meat quality trait. The callipyge gene promotes muscle growth, but inhibits fat deposition and reduces tenderness. Additionally, heat shock proteins have an inhibitory effect on tenderness, while maintaining color stability.
iv. The accuracy and preference of multi-omics technologies somewhat affect their authenticity. Currently, after optimizing the data acquisition methodology, detection coverage was enhanced during experimental validation, though some transcripts, proteins, and metabolites with lower expression abundances remained undetected, resulting in a lack of complete data in the multi-omics databases.
v. Efficient and scientific methods are still needed for correlating and integrating the datasets from multidimensional omics studies of transcriptomes, proteomes, and metabolomes.
vi. There is an insufficient crossover between differentiated disciplines. It is necessary to perform differentiation and synthesis to ensure high-quality meat production.

### 6.3. Directions for Addressing These Challenges

i. Strengthening the application of tracer technology is crucial for studying sheep and goat meat quality and elucidating the regulatory mechanisms. Attention should be paid to intracellular substance dynamics to better explain the mechanisms related to meat quality.
ii. When multi-omics technologies are used to explore meat quality mechanisms, potential molecular biomarkers require validation in a reasonable manner.
iii. The exploration of biomarkers for individual meat quality attributes should be emphasized to facilitate the potential targeted control of meat quality.
iv. High-throughput multi-omic technologies can verify each other. State-of-the-art omics technologies, including third-generation transcriptomics, ‘4D’ proteomics, and spatial omics, enable rapid identification, deep coverage, and high accuracy. Applying these advanced techniques in future meat quality studies is recommended.
v. A multi-omics database with genes as connection points should be established, along with integration algorithms and software for multi-omics data.
vi. Utilizing interdisciplinary knowledge in research on sheep/goat biomarkers related to meat quality can improve breeding evaluation accuracy, reduce the associated costs, and accelerate the breeding process. Combining this with proper feeding, slaughter, transportation, and processing management has great potential to produce high-quality meat.
